# Physiological and Microstructure Analysis Reveals the Mechanism by Which Formic Acid Delays Postharvest Physiological Deterioration of Cassava

**DOI:** 10.3390/antiox13101245

**Published:** 2024-10-16

**Authors:** Yannian Che, Zhongping Ding, Chen Shen, Alisdair R. Fernie, Xiangning Tang, Yuan Yao, Jiao Liu, Yajie Wang, Ruimei Li, Jianchun Guo

**Affiliations:** 1 School of Life Sciences, Hainan University, Haikou 570228, China; 22110710000011@hainanu.edu.cn (Y.C.); 21220951310010@hainanu.edu.cn (Z.D.); 22220860000067@hainanu.edu.cn (X.T.); 2 National Key Laboratory for Tropical Crop Breeding, Institute of Tropical Bioscience and Biotechnology, Chinese Academy of Tropical Agricultural Sciences, Haikou 571101, China; 2023301120122@webmail.hzau.edu.cn (C.S.); yaoyuan@itbb.org.cn (Y.Y.); liujiao@itbb.org.cn (J.L.); wangyajie@itbb.org.cn (Y.W.); 3 Sanya Research Institute, Chinese Academy of Tropical Agricultural Sciences, Sanya 572024, China; 4 College of Plant Science & Technology, Huazhong Agricultural University, Wuhan 430070, China; 5 Root Biology and Symbiosis, Max Planck Institute of Molecular Plant Physiology, Am Mühlenberg 1, 14476 Potsdam, Germany; fernie@mpimp-golm.mpg.de

**Keywords:** formic acid, shelf life, cassava, postharvest, cell wall, pectin

## Abstract

Formic acid is reported to act as a food preservative and feed additive, but its effects on controlling postharvest physiological deterioration (PPD) development in cassava are unclear. In this study, we assessed the effectiveness of different concentrations of formic acid in attenuating PPD occurrence in fresh-cut cassava. The results showed that the concentration of 0.1% (*v*/*v*) formic acid could significantly delay the occurrence of PPD, and that the higher the concentration of formic acid supplied, the later the occurrence of PPD symptoms. The physiological and biochemical analysis of 0.5%-formic-acid-treated cassava slices revealed that formic acid decreased the degradation of starch, inhibited the accumulation of hydrogen peroxide (H_2_O_2_), malondialdehyde (MDA), and water-soluble pectin in cassava slices with PPD development, and increased the activities of the antioxidant enzymes ascorbate peroxidase (APX) and glutathione reductase (GR). A microscopic observation showed that the formic acid treatment inhibited the enlargement of the intercellular space during the cassava PPD process, which suggests that the formation of an intercellular layer of the cell wall was inhibited by formic acid. This study thus revealed the mechanism used by formic acid to extend the cassava shelf life; however, a detailed evaluation of the possible side effects on, for example, the cyanide content will be needed to categorically ensure the safety of this method.

## 1. Introduction

The starch-rich cassava (*Manihot esculenta* Crantz) tuberous roots are an important source of human food, animal feed and industrial bioenergy. Considering the advantages of cassava in various applications, the demand for cassava is increasing day by day. However, cassava is prone to postharvest physiological deterioration (PPD), which generally starts within 12–72 h after harvest symptoms appear, resulting in discoloration and a bad taste, with the starch quality additionally being seriously impaired [1,2]. The activation and intensity of PPD in cassava is caused by mechanical damage, as evidenced by the first occurrence of PPD symptoms at the injured site. The injuring of the cassava triggers an increase in the respiratory rate, the accumulation of reactive oxygen species (ROS), and the production of cyanide (which is toxic to animals) [3,4]. After mechanical injury, the production of hydrogen peroxide significantly increases within 15 min. Within 4 h after harvest, the malondialdehyde (MDA) content increases in the regions closest to the injury site [5]. The rapid PPD of cassava is a limiting factor in cassava production, leading to significant postharvest losses of up to 29% globally [6]. Therefore, delaying the occurrence of PPD in cassava has become an important issue that producers urgently need to solve.

Formic acid is the smallest organic acid and the precursor for amino acids; therefore, it plays significant roles in organic life. In agricultural production, formic acid is used as a food preservative, feed additive, and non-antibiotic growth promoter [7,8,9]. The formic acid content decreases gradually during the storage of tofu, and is used as an indicator of tofu freshness [10]. In the process of silage production, the addition of formic acid not only improves the quality of the silage, but also extends its shelf life [8,11,12]. Using feed additives containing formic acid can improve the growth performance of weaned piglets [13]. Feeding diets containing formic acid could regulate intestinal growth and improve the disease resistance of gilthead sea bream [14]. Formic acid fumigation greatly reduces the rot rate of citrus fruits [15]. Formic acid is also used for chitosan dissolution and is used to control the postharvest decay of strawberries and table grape [16,17]. Formic acid inactivates *Salmonella* and maintains the postharvest quality of cherry tomatoes [18]. Formic acid can inhibit the growth of *Bacillus subtilis*, which causes the postharvest spoilage of potatoes, thereby preventing the deterioration and discoloration of potatoes during storage [7]. A mixture of formic acid and levulinic acid is used to control postharvest citrus blueberry disease [19]. However, the effectiveness of formic acid in controlling the PPD of cassava has not been studied.

The objective of this study was to evaluate whether formic acid can control the occurrence of PPD in cassava slices. Another objective is to analyze the potential mechanisms of formic acid in controlling the PPD of cassava.

## 2. Materials and Methods

### 2.1. Plant Materials and Treatments

The cassava utilized in this study was harvested from bitter cassava variety SC8 plants grown in a field for 6 months. Immediately after harvest, the cassava plants were transported to the laboratory for experimental use. The cassavas were washed with tap water and ultrapure water in turn and then drained using tissue paper. The cleaned cassavas were evenly cut into 5 mm slices and dipped in deionized water (for control) and 0.1%, 0.3%, 0.5%, 1%, 2%, and 5% (*v*/*v*) formic acid for 2 h. Subsequently, the treated cassava slices were rinsed with deionized water, drained, and placed in a plastic storage box with a temperature of 26 °C and a relative humidity of 70%. The changes in the sections of each cassava slice were observed and photographed at 0 h, 6 h, 12 h, 24 h, and 48 h after storage to evaluate the PPD occurrence. Three slices per sample of 0.5%-formic-acid-treated cassava at the 0 h, 6 h, 12 h, 24 h, and 48 h time points were collected and rapidly frozen with liquid nitrogen and then frozen at −80 °C for subsequent utilization.

### 2.2. Starch Content Measurement

The starch content of the cassava slices treated with 0.5% formic acid and the control after storage for different durations (0, 6, 12, 24, and 48 h) was measured by the anthrone colorimetric method using a plant starch content kit (Comin, Suzhou, China). According to the manufacturer’s instructions, the cassava samples were homogenized with 80% ethanol and extracted at 80 °C in a water bath for 30 min, and then centrifuged at 25 °C and 3000× *g* for 5 min. The precipitate was suspended with distilled water, gelatinized in a 95 °C water bath for 15 min, and then extracted with 9.2 mol/L HClO_4_ and diluted with distilled water. Subsequently, the mixture of supernatant and 2% anthrone was heated in a boiling water bath for 10 min. After cooling down, the mixture was used to measure the absorbance value at a 620 nm wavelength. The starch content was expressed in mg/g. Three biological replicates were performed.

### 2.3. H_2_O_2_ Content Analysis

The hydrogen peroxide (H_2_O_2_) content of cassava slices treated by 0.5% formic acid and control after storage for different time points (0, 6, 12, 24, and 48 h) was determined based on the titanium sulfate method. Briefly, the cassava samples were homogenized in with 4 °C precooled acetone and centrifuged to obtain the supernatant. The supernatant was mixed with 5% titanium sulfate and concentrated ammonia to obtain the precipitate. The precipitate was then washed with acetone and dissolved with 2 mol/L sulfuric acid. After that, 200 μL liquid was used to determine the absorption value at a 415 nm wavelength. The H_2_O_2_ content was expressed in μmol/g FW. Three biological replicates were performed of H_2_O_2_ content measurement.

### 2.4. Determination of Malondialdehyde Content

The malondialdehyde (MDA) content in cassava was measured based on the thiobarbituric acid method. The cassava samples were homogenized in liquid nitrogen and extracted with 10% TCA solution. The supernatant was mixed with 0.5% TBA and heated at 90 °C for 30 min. The absorbance at 532 nm and 600 nm of supernatant was measured. The MDA content was expressed in nmol/g FW. Three biological replicates were performed for the MDA content measurement.

### 2.5. Ascorbate Peroxidase Activity

The ascorbate peroxidase (APX) activity was determined using APX activity kit (Comin, Suzhou, China), according to the manufacturer’s instructions. An amount of 0.1 g of the sample was weighed and added into 1 mL of 50 mmol/L K_2_HPO_4_-KH_2_PO_4_ (pH7.0) for ice bath homogenization. The mixture was centrifuged at 4 °C and 13,000× *g* for 20 min to obtain the supernatant. The mixture of 20 μL of supernatant, 140 μL of K_2_HPO_4_-KH_2_PO_4_ (pH 7.0), 20 μL of 0.3 mmol/L AsA, and 0.06 mmol/L H_2_O_2_ was used to measure the 10 s absorbance (A1) and 130 s absorbance (A2) at a 290 nm wavelength. The APX activity was calculated using the following formula: APX (μmol/min/g FW (fresh weight)) = 3571 × (A1 − A2) ÷ W (sample weight).

### 2.6. Glutathione Reductase Activity

The glutathione reductase (GR) activity was determined using GR activity kit (Comin, Suzhou, China), according to the manufacturer’s instructions. About 0.1 g of the sample was weighed and added into 10 mL of 4 °C precooled 100 mmol/L phosphoric acid buffer (including 1 mmol/L EDTA, pH 7.5) for ice bath homogenization. Subsequently, the mixture was centrifuged at 4 °C and 8000× *g* for 15 min, and the supernatant is the crude enzyme. Next, 150 μL of 100 mmol/L phosphoric acid buffer (including 1 mmol/L EDTA, pH 7.5), 20 μL of 5 mmol/L GSSG solution, 20 μL of crude enzyme liquid, and 10 μL of 4 mmol/L NADPH solution were added to 96-well plates which were mixed immediately. The initial absorbance (A1) and 180 s absorbance (A2) of supernatant were quickly determined at a 340 nm wavelength. The GR activity was calculated using the following formula: GR activity (nmol/min/g FW) = 1072 × (A1 − A2) ÷ W (sample weight).

### 2.7. Water-Soluble Pectin Content Measurement

To measure the water-soluble pectin (WSP) content, about 0.3 g of the sample was weighed, and 1 mL of 80% ethanol was added prior to rapid homogenization at room temperature; next, the mixture was placed in a water bath at 95 °C for 20 min, and then cooled to room temperature and centrifuged at 25 °C and 4000× *g* for 10 min, and the supernatant was then discarded. The precipitate was subsequently washed with 1.5 mL of 80% ethanol and 1.5 mL of 80% acetone one by one (these solutions were swirled for about 2 min and then centrifuged at 25 °C and 4000× *g* for 10 min, and the supernatant was then discarded). Next, 1 mL 63% ethanol was added to the precipitate (in order to remove starch) and soaked for 15 h, prior to centrifuging at 4000× *g* and 25 °C for 10 min, and then the supernatant was discarded and the precipitate was dried. Next, 3 mg of dried precipitate was fully homogenized in 1 mL of distilled water and the homogenate was centrifuged at 4 °C and 8000× *g* for 10 min with the supernatant representing WSP extract. Subsequently, 50 μL of WSP extract was mixed with 50 μL of 0.1% carbazole reagent and 400 μL of concentrated sulfuric acid. The mixture was heated at 85 °C for 5 min. Next, 200 µL of the solution was taken to measure the absorbance value at a 530 nm wavelength. The galacturonic acid was used for standard curve preparation. The mixture of distilled water, carbazole reagent, and concentrated sulfuric acid was measured as a blank. The WSP content was expressed in mg/g DW (dry weight).

### 2.8. Microscopic Observation

The cassava samples were stored for 6 h after treatment with (0.5%) or without formic acid, and control (fresh cassava) samples were cut into 1–2 mm^3^ small blocks and fixed by microscope fixation solution (Servicebio, Wuhan, China). The tissues were embedded with resin 812, and then ultra-thin sections were produced using an ultra-microtome (Leica UC7). The microstructure were observed and photographed using a HITACHI HT7800 transmission electron microscope.

### 2.9. Statistical Analysis

All data are represented as means ± SEM of three replicates. A statistical analysis was conducted using GraphPad Prism 8.0 software (San Diego, CA, USA) with one-way analysis of variance (ANOVA) and Dunnett’s multiple comparisons tests for measuring significance. *p* < 0.05 was considered statistically significant.

## 3. Results

### 3.1. Formic Acid Treatment Maintained the Appearance of Cassava Slices

**Figure 1:**
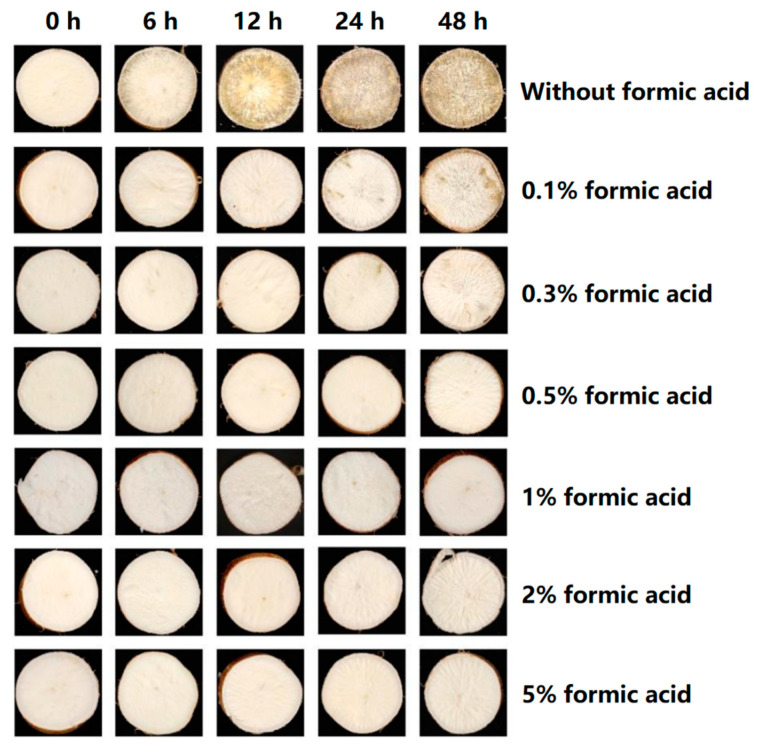
Effect of formic acid in delaying cassava postharvest physiological deterioration (PPD). Visual examination of cassava slices at different time points during storage (0, 6, 12, 24, and 48 h) after incubation with different formic acid solution (0.1%, 0.3%, 0.5%, 1%, 2%, and 5% (*v*/*v*)) for 2 h.

Visual observations indicated that the CK-treated group (without formic acid) of fresh-cut cassava began to generally appear black and brown stripes within 6 h of storage at 26 °C and 70% relative humidity condition (Figure 1). Moreover, with the extension of storage time, the browning degree of the surface of the slices was deepened. The slight browning was not observed in the 0.1%-formic-acid-treated group until 24 h storage, and the browning was delayed with the increase in formic acid concentration. When the concentration of formic acid was 0.5%, no browning occurred in the root slices 48 h after treatment (Figure 1). Subsequently, we chose the samples treated with 0.5% formic acid at different time points for further study.

### 3.2. Formic Acid Inhibited the Degradation of Starch during Storage

**Figure 2:**
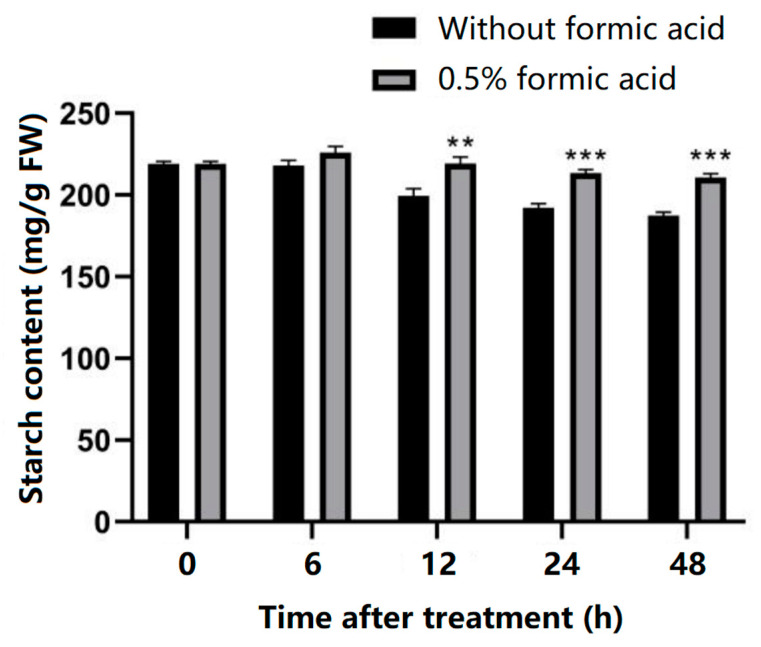
Effect of formic acid on starch content during storage. ** indicates the significant difference *p* ≤ 0.01, *** indicates the significant difference *p* ≤ 0.001.

Starch content is an important quality indicator of cassava after harvest. The occurrence of PPD can lead to the degradation of starch, so this study investigated whether formic acid affects the change in starch content during the occurrence of PPD in cassava. The results show that the starch content of cassava slices in control group decreased gradually with the extension of storage time. While the starch content of the 0.5%-formic-acid-treated group was significantly higher than that in the control group at 12, 24, and 48 h (Figure 2). These results indicate that formic acid reduces starch degradation in the cassava slices during storage, thereby maintaining the quality of cassava after harvest.

### 3.3. Formic Acid Inhibited the Accumulation of H_2_O_2_

**Figure 3:**
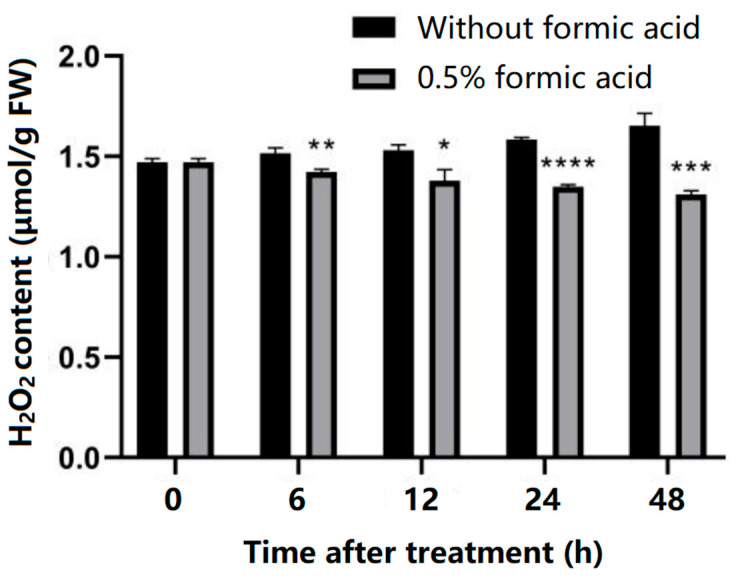
Effect of formic acid on reactive oxygen species (ROS) accumulation during cassava PPD occurrence. H_2_O_2_ content was determined in cassava slices with (0.5% *v*/*v*) or without formic acid treatment during PPD occurrence. * indicates the significant difference *p* ≤ 0.1, ** indicates the significant difference *p* ≤ 0.01, *** indicates the significant difference *p* ≤ 0.001, **** indicates the significant difference *p* ≤ 0.0001.

Reactive oxygen species (ROS) accumulation in cassava slices of the treated group and control group during PPD was analyzed by measuring hydrogen peroxide (H_2_O_2_) content. As shown in Figure 3, with the extension of storage time, the H_2_O_2_ content of cassava slices in the control group gradually increased, while the H_2_O_2_ content of cassava slices in the treated group showed a slow trend of decline, and the H_2_O_2_ content was significantly lower than that in the control group, indicating that formic acid treatment reduced ROS accumulation in the cassava PPD process.

### 3.4. Formic Acid Inhibited the Accumulation of MDA

**Figure 4:**
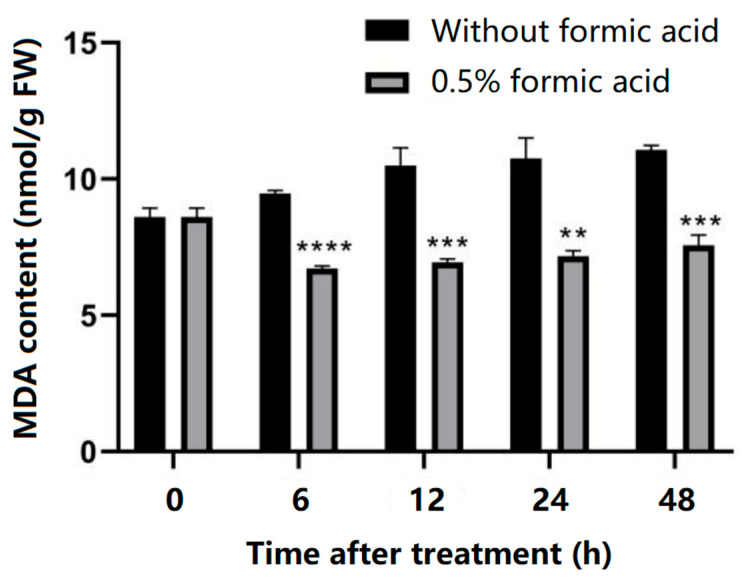
Effect of formic acid on membrane integrity during cassava storage. Malondialdehyde (MDA) content was determined in cassava slices with (0.5% *v*/*v*) or without formic acid treatment during cassava storage. ** indicates the significant difference *p* ≤ 0.01, *** indicates the significant difference *p* ≤ 0.001, **** indicates the significant difference *p* ≤ 0.0001.

The MDA content of cassava slices in the treated group and the control group was determined, and it was found that the MDA content of cassava slices in the control group gradually increased with the extension of storage time. Although the MDA content of cassava slices in the treated group also increased slowly with the extension of storage time, it was significantly lower than that in the control group at 6 h, 12 h, 24 h, and 48 h (Figure 4), indicating that the membrane lipid peroxidation degree of the slices in the treated group was lower than that in the control group during PPD, and the membrane suffered less peroxidation damage.

### 3.5. Formic Acid Enhance APX and GR Activities

**Figure 5:**
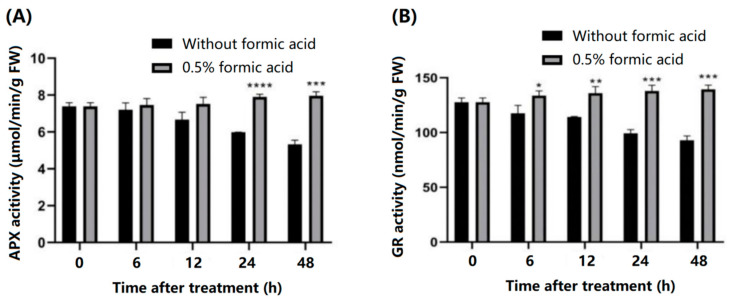
Effect of formic acid on the activities of ascorbate peroxidase (APX) and glutathione reductase (GR) during cassava slices storage. (**A**) APX enzyme activities in cassava slices with (0.5% *v*/*v*) or without formic acid treatment during PPD occurrences. (**B**) GR activities enzyme activities in cassava slices with (0.5% *v*/*v*) or without formic acid treatment during PPD occurrences. * indicates the significant difference *p* ≤ 0.1, ** indicates the significant difference *p* ≤ 0.01, *** indicates the significant difference *p* ≤ 0.001, **** indicates the significant difference *p* ≤ 0.0001.

To further clarify whether glutathione reductase (GR) and ascorbate peroxidase (APX) are directly involved in the regulation of PPD symptoms and their association with formic acid in ROS clearance, we measured the enzymatic activity of these two enzymes during PPD development in cassava slices. It was found that the activities of APX and GR decreased gradually with the extension of storage time in cassava slices of control group, while the activities of APX and GR increased slowly with the extension of storage time in the cassava slices of the treated group. Moreover, the APX enzyme activity in the 0.5%-formic-acid-treated group was significantly higher than that in the control group at 24 h and 48 h, and the GR activity in the treated group at 6 h, 12 h, 24 h, and 48 h was significantly higher than that in the control group (Figure 5). These results indicated that the activated oxygen scavenging ability of the formic acid-treated group was enhanced, and the structural integrity of the biofilm system (such as plasma, nuclear membrane, and mitochondria membrane) was better than that in the control group during PPD. The ability of defense membrane lipids to peroxidation is stronger.

### 3.6. Changes in Water-Soluble Pectin Content

**Figure 6:**
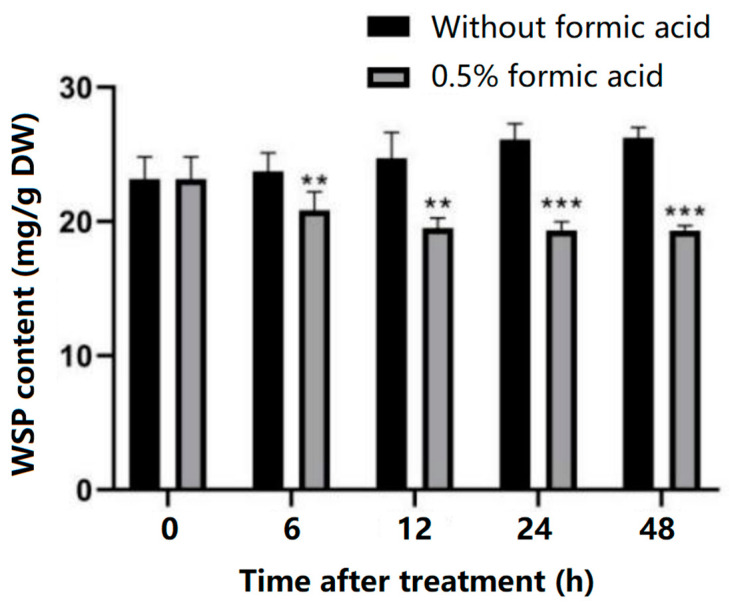
Effect of formic acid on the water-soluble pectin (WSP) content during cassava PPD development. ** indicates the significant difference *p* ≤ 0.01, *** indicates the significant difference *p* ≤ 0.001.

The water-soluble pectin (WSP) content of cassava slices in the treated group and the control group was measured. It was found that the WSP content of cassava slices in the control group gradually increased with the extension of storage time, while the WSP content in the treated group began to decrease at 6 h and tended to be stable at 12 h, 24 h, and 48 h. The WSP content of cassava slices in the treated group was significantly lower than that in the control group at 6 h, 12 h, 24 h, and 48 h (Figure 6), indicating that the softening degree of cassava slices in the PPD process was lower than that in the control group, which assisted to confirm that the degree of PPD in the treated group was lower than that in the control group.

### 3.7. Changes in Microstructure

**Figure 7:**
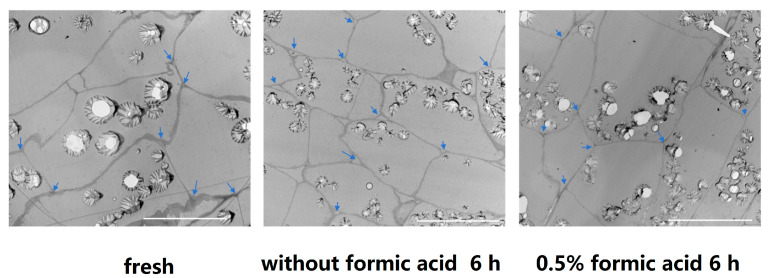
Effect of formic acid on microstructure of cassava slices 6 h after treatment. Control: fresh-cut cassava; without formic acid 6 h: 6 h after treatment without formic acid; 0.5% formic acid 6 h: samples of 6 h after treatment by 0.5% formic acid. The blue arrows point to the intercellular space formed by three cells. The scale bar indicates 50 μm.

To investigate whether formic acid effects the cell microstructure, we performed microscopic observations on cassava slices of 6 h after treatment with (0.5%) or without formic acid. The result showed that, compared with the fresh cassava, in the cassava sample at 6 h after treatment without formic acid, the intercellular space was enlarged, indicating that the intercellular layer of the cell wall was degraded. By comparison, 6 h after treatment with 0.5% formic acid, the intercellular space was still as small as that in fresh cassava, indicating that the intercellular layer of the cell wall was not degraded (Figure 7). These results suggested that formic acid suppressed the cell wall degradation, leaving the cells in an intact and stable state and thus not susceptible to deterioration.

## 4. Discussion

As an important food, feed, and biomass energy raw material, cassava plays an crucial role in human life and production. PPD is the main cause of cassava postharvest loss of quality and yield. Multiple chemicals have been tested to control PPD in cassava, such as ethanol [20], lysozyme [21], ethephon [22], acetic acid [23], and chitosan [24]. Formic acid has been confirmed to control food, fruit, and feed storage quality [7,8,10,11,16,18]. However, the impact of formic acid on the PPD process of cassava remains unexplored. In this study, we used formic acid to treat fresh-cut cassava to verify its effects on controlling PPD. The results showed that formic acid has a significant inhibitory effect on PPD in cassava, and the onset of PPD symptoms was postponed even when treated at a low concentration (0.1% *v*/*v*) (Figure 1). PPD often leads to the degradation of starch [20]. In this study, the decreased starch content was observed in control cassava during storage, and 0.5% formic acid prevented the decline in starch content (Figure 2). These results confirm the effectiveness of formic acid in delaying the postharvest deterioration of cassava.

The accumulation of ROS has been revealed to be one of the main reasons for PPD, of which H_2_O_2_ is a common substance being valuated. In this study, the H_2_O_2_ content increased during the storage period in control cassava, and formic acid significantly suppressed ROS accumulation (Figure 3). The accumulation of ROS in vivo will lead to membrane lipid peroxidation in plants, and the final product of oxidation is malondialdehyde (MDA). Therefore, the determination of MDA content can reflect the degree of membrane damage by peroxide. Its content has been widely used as a marker to assess the degree of oxidative stress damage to plants [25]. In this study, the MDA content increased gradually in control cassava during storage, while that in the formic acid-treated cassava was significantly reduced (Figure 4). The suppression of both H_2_O_2_ and MDA content in delaying cassava PPD occurrence has also been observed in a previous study [22].

Ascorbate peroxidase (APX) is the most important peroxidase in H_2_O_2_ detoxification, it has a greater affinity for H_2_O_2_ than catalase [26]. Previous studies have found that increased APX activity is positive to preserve fruit quality during postharvest storage [27,28,29]. The increased APX activity was also associated with delayed PPD occurrence in cassava, which is similar to this study [30]. In this study, formic acid significantly promoted the increase in the APX activity during storage, making cassava free of PPD (Figure 5A). This study further confirmed the relationship between APX and PPD in cassava. Glutathione reductase (GR) is another important enzyme in H_2_O_2_ detoxification in plants. It belongs to the same ROS scavenging pathway as APX in the ascorbate–glutathione (AsA-GSH) cycle. Numerous studies have found that inducing increased GR activity is consistent with maintaining fruit quality during storage [26,31,32]. In cassava, melatonin delayed cassava PPD by increasing the GR activity, but not the APX activity [33]. In this study, formic acid significantly increased the GR and APX activity, suggesting that formic acid may delay cassava PPD by inducing the AsA-GSH cycle pathway to clear H_2_O_2_.

Previous studies have found that there is a certain correlation between cell wall structure changes and the PPD of fresh products [34]. In the process of postharvest browning of potato, the intercellular layer of the cell wall was partially degraded and the intercellular space increased [35]. The collapsed cell wall structure was also observed in grapes when panicle browning occurred [36]. In this study, formic acid inhibited the intercellular layer degradation during cassava storage, keeping the intercellular space as small as in fresh cassava. The intercellular layer of the cell wall is mainly composed of complex pectin substances, and the increase in water-soluble pectin (WSP) content is due to the degradation of intercellular layer and the destruction of the cell wall structure [34,37]. During storage, the water-soluble pectin (WSP) increased along with PPD; however, when the occurrence of PPD was delayed by some treatments, the increase in the WSP content was also inhibited in many kinds of fresh agriculture products, such as litchi [38], grape [36], banana [39], *Zizania latifolia* [40], and tomato [41]. In this study, formic acid inhibited the increase in the WSP content, indicating that formic acid can maintain the integrity of the cell wall by inhibiting the increase in the WSP content and reducing the degradation of the intercellular layer, so as to delay the occurrence of PPD.

People may be confused about the use of formic acid because it is toxic in high concentrations. However, formate is a central metabolite, and its addition will as such be unlikely to have major metabolic deleterious consequences [42]. It is found naturally in many foods such as honey, wine, coffee fruit, milk, cheese, and yogurt [7]. Ingesting normal levels of formic acid is easily and quickly metabolized by the human body without causing harm [42]. Formic acid is approved as a component of adjuvants and synthetic flavorings to be directly added into food for human use, as a component of paper materials for food wrapping, as a flavoring, and as a preservative agent [7]. Formic acid can be used in foods and products provided it does not pose significant health concerns. It is also utilized as an additive in animal food and drinking water [7]. The accepted daily intake for human consumption is 0–3 mg/kg [7]. The European Food Safety Authority classifies formic acid as safe, with no restrictions on its use in food [7]. The purpose of the application of formic acid in this study is only to study the internal mechanism controlling the occurrence of PPD in cassava. Its addition and application in food should be carried out in accordance with regulations.

According to a previous study, mechanical injury during cassava harvest does lead to the production of cyanide, which is toxic to human and animal [43]. However, the relationship between cyanide and PPD remains controversial. For instance, cyanide content decreased during cassava postharvest storage [3,44]. The hot water dip delayed cassava PPD occurrence, but increased the cyanide (hydrocyanic acid) content [45]. These studies suggested that high cyanide content can delay cassava PPD. However, in another study, the cassava genotypes containing a low level of cyanide showed less PPD than genotypes containing a high level of cyanide [46]. Moreover, in a study on coating methods to retain the cassava postharvest quality, the cyanide content decreased compared to that in the control [3]. Therefore, we did not detect the cyanide in this study. As a C1 metabolite itself, formic acid is involved in the metabolism of many substances, such as polyamine, sterol, formaldehyde, folate, tryptophan, and purine [42]. Whether formic acid is involved in the synthesis of cyanide and whether the exogenous use of formic acid affects the synthesis of cyanide remains to be carefully investigated.

## 5. Conclusions

This study provides information on the use of formic acid in cassava slicing to delay PPD occurrence. In fact, the use of formic acid helps to significantly extend shelf life by increasing APX and GR enzyme activity, reducing H_2_O_2_ and MDA content, and discoloration. In addition, formic acid also inhibited the degradation of intercellular layer by reducing the content of WSP, maintaining the stability of cell wall, inhibiting the occurrence of PPD, and inhibiting the degradation of starch, thus maintaining the quality of cassava during storage. The results of this study provide a method for effectively delaying the occurrence of PPD in cassava, and also provide a direction for future targeted improvement of cassava storage quality. However, whether formic acid affects cassava cyanide content is unclear and needs further investigation in the future.

## Data Availability

The datasets generated during and/or analyzed during the current study are available from the corresponding author on reasonable request.
